# A survey on knowledge and self-reported formula handling practices of parents and child care workers in Palermo, Italy

**DOI:** 10.1186/1471-2431-9-75

**Published:** 2009-12-10

**Authors:** Giuseppe Calamusa, Rosalia Maria Valenti, Ivana Guida, Caterina Mammina

**Affiliations:** 1 Department of Sciences for Health Promotion "G D'Alessandro", Section of Hygiene, University, Via del Vespro 133, I-90127 Palermo, Italy; 2 PhD School in "Alimentazione e nutrizione umana", University, Palermo, Italy

## Abstract

**Background:**

Powdered infant formula (PIF) is not a sterile product, but this information appears to be poorly diffused among child caregivers. Parents and child care workers may behave in an unsafe manner when handling PIF.

**Methods:**

This study involved parents and child care workers in the 24 municipal child care centres of Palermo. Knowledge and self-reported practices about PIF handling were investigated by a structured questionnaire. A Likert scale was used to measure the strength of the respondent's feelings. Association of knowledge and self-reported practices with demographic variables was also evaluated.

**Results:**

42.4% of parents and 71.0% of child care workers filled in the questionnaire. Significant differences were found between parents and child care workers for age and education. 73.2% of parents and 84.4% of child care workers were confident in sterility of PIF. Generally, adherence to safe procedures when reconstituting and handling PIF was more frequently reported by child care workers who, according to the existing legislation, are regularly subjected to a periodic training on food safety principles and practices. Age and education significantly influenced the answers to the questionnaire of both parents and child care workers.

**Conclusion:**

The results of the study reveal that parents and child care workers are generally unaware that powdered formulas may contain viable microorganisms. However, child care workers consistently chose safer options than parents when answering the questions about adherence to hygienic practices.

At present it seems unfeasible to produce sterile PIF, but the risk of growth of hazardous organisms in formula at the time of administration should be minimized by promoting safer behaviours among caregivers to infants in both institutional settings and home.

## Background

Powdered infant formula (PIF) is not a sterile product and opportunistic pathogens could multiply in the reconstituted product resulting in neonatal infections [1-4]. Several surveys have provided an overview of the pathogens that may be isolated in PIF [1,3]. In 2006, an expert meeting organized by the Food and Agriculture Organization of the United Nations (FAO) and the World Health Organization (WHO) concluded that the microorganisms of greatest concern in PIF are *Salmonella enterica *and *Cronobacter *spp. (previously *Enterobacter sakazakii) *[2,5]. However, recently, some reports have suggested a possible pathogenic role for other members of *Enterobacteriaceae*, i.e. *Pantoea *spp. and *Enterobacter hormaechei *that have been similarly detected in unopened PIF [6,7].

The incidence of infection with foodborne pathogens is significantly higher in children under one year of age than in older age groups [8]. This could be at least partially justified by a physiologically increased vulnerability and a higher probability among parents and caregivers to seek care and among health professionals to report illness in infants vs. older children. However, infants who are not breastfed have proven to be at increased risk for some foodborne infections, such as salmonellosis and diarrhea of viral etiology [9,10]. Moreover, in industrialized countries socio-demographic factors, such as unemployment and low level of education of parents, appear to play a dual role of risk factors for bacterial enteric infections in children, but also for less frequent and shorter breastfeeding [10,11]. WHO recommends that infants should be exclusively breastfed for the first 6 months of life to achieve optimal growth and health, but this is not always feasible, and thus, infants who are being not breastfed require appropriately formulated infant nutritional products [12-14]. In Italy in 1998, an estimated 15% of newborns were given PIF at discharge and up to 90% at five months [13]. A mean annual consumption of about 11.7 kg of PIF per infant was estimated in 2004 [15].

Unopened PIF is not sterile, but depending on storage, handling and feeding practices, there is potential for microbial growth in reconstituted formula, amplifying what would otherwise be a very low contamination level. Accordingly, WHO and FAO have recently issued new guidelines for the safe preparation, storage and handling of PIF [16].

The domestic environment and child care centres are the two main areas of possible exposure to contaminated PIF for healthy infants and children. Several reports show that hygienic food handling behaviours of both professional food workers and consumers are frequently inadequate [17,18]. Particular concern arises from handling formula and associated equipment in the domestic kitchen environment that could be contaminated with foodborne pathogens or potentially harmful opportunistic organisms [19].

The objective of this study was to assess and compare knowledge and self-reported formula handling practices of a sample of parents and child care workers living in Palermo, Italy, against the WHO/FAO guidelines. Association with some socio-demographic variables was also evaluated.

## Methods

### Setting

The study was carried out in Palermo on parents and child care workers recruited in April 2008 in all the 24 municipal child care centres of Palermo. These centres are evenly distributed in the urban area and offer day care, including supervised indoor and outdoor activities as well as meals and snacks, to children under the age of three. All infants and children are entitled to attend municipal day care centres, but priority is given to low income families. Between 30 and 50 children have annual access to each municipal facility.

### Methods

A structured questionnaire was designed and self-administered to the parents and child care workers. Parents were asked to participate in the study by a written invitation from the child care workers of each centre, who had been previously informed about the objectives of the investigation and the instructions to be given. One parent only per child was allowed to fill in the questionnaire. All the child care workers were also invited to participate to the investigation by completing the same questionnaire. Since 1997, the child care centre staff is subjected according to the recent European legislation (Directive 93/43/EEC and Regulation EC 852/2004) to a mandatory periodic training in the form of formal courses pertaining to food safety and good hygienic practices, but without specific instructions on safe formula handling.

Full information on the study and its purposes was provided to participants. Completion of the questionnaire implied respondent consent to participate in the study. According to the Italian regulations, ethical approval was not required for this study.

The questionnaire consisted of a first section containing demographic questions, which included gender, race or ethnicity, education level and number of children. The second section contained 13 questions about awareness of the likelihood that microrganisms are in the formula and self-reported formula handling practices as compared to the procedure recommended by the WHO/FAO guidelines [16]. For this portion of the survey, a Likert scale was used to measure the strength of the respondent's feelings. For each statement, a score of 1, 2, 3, 4 or 5 was assigned to responses of strongly agree, agree, no opinion, disagree or strongly disagree, respectively, without scoring reversed for negatively worded statements.

In finalizing the questionnaire, the comments and comprehension difficulties of several people, some who were involved in the project and others who were not, were taken into account. An informal pilot survey was preliminarily conducted among the personnel of the Department of Sciences for Health Promotion to identify issues of timing, wording or routeing errors. The pilot-interviewed subjects did not necessarily reflect the characteristics of the target population.

EpiInfo software ver. 6.0 (CDC, Atlanta, GA, US) was used for data management and analysis. For descriptive statistics, analysis of frequency and central tendency and dispersion (e.g. means and standard deviations, SDs) were calculated to describe demographic characteristics and formula handling knowledge and self-reported practices. The answer patterns obtained from parents and child care workers in the second section of the questionnaire were evaluated by calculating the mean scores for each question. In the subsequent statistical analysis of the association between demographics and answers to the questionnaire, age, education level and number of children were entered as categorical variables: respectively, younger, ≤34 years and older, ≥35 years; low, ≤8 years and high, >8 years; and one or two or more children. Cut-offs were chosen based upon the demographic profile of the population under study, the mandatory level of education in Italy (i.e. 8 years), when the participants started attending their primary school, and previous reports suggesting that having more than one child could significantly affect some food safety behaviours [20].

One-way analysis of variance (ANOVA or Kruskall-Wallis, when appropriate) and cross tabulation with chi-square statistics were used to evaluate the relationship between knowledge, self-reported practices and each demographic variable. In all analyses, a α level of 0.05 or below was indicative of a statistically significant difference.

## Results

Of the 983 parents invited to participate, 417 (42.4%) completed the survey, whereas 314 (71.0%) out of the 442 child care workers filled in the questionnaire. Table 1 provides a statistical description of the respondents in the study.

**Table 1:** Demographic characteristics of survey respondents.

	Parents		Child care workers	
		**% of respondents**		**% of respondents**

Total	*n *= 417		*n *= 314	

				

Age (yr)*				

<35		44.6		9.0

35-44		51.8		45.8

≥45		3.6		45.2

Total	*n *= 392	100	*n *= 299	100

Mean (SD)		35.09 (5.23)		44.04 (6.85)

Unanswered	*n *= 25 (6.0%)		*n *= 15 (4.8%)	

				

Ethnicity*				

Italian		93.5		99.7

Other		6.5		0.3

Total	*n *= 401	100	*n *= 306	100

Unanswered	*n *= 16 (4.0%)		*n *= 8 (2.6%)	

				

Education (yr)*				

<5		0.5		0

5		4.9		1.6

8		26.4		20.8

13		49.1		68.7

>13		19.1		8.9

Total	*n *= 402	100	*n *= 303	100

Unanswered	*n *= 15 (3.6%)		*n *= 11 (3.5%)	

				

Gender†				

Male		9.2		4.8

Female		90.8		95.2

Total	*n *= 414	100	*n *= 314	100

Unanswered	*n *= 3 (0.7%)		*n *= 0	

				

Nr of children*				

0		0		7.4

1		33.6		24.2

2		45.6		51.3

>2		20.6		17.1

Total	*n *= 399	100	*n *= 269	100

Mean (SD)		1.94 (0.94)		1.80 (0.86)

Unanswered	*n *= 18 (4.3%)		*n *= 45 (14.3%)	

* P < 0.001

† P = 0.01

Statistically significant differences were detected for all the demographic variables under consideration between parents and child care workers, with respondents within parents being more likely younger and with 8 years or less of education (Table 1).

Table 2 in the Additional File 1 presents the detailed results of the analyses for awareness of possible presence of microorganisms in PIF and self-reported practices for the preparation, handling, administration and storage of PIF and comparison between parents and child care workers. Seventy three-two percent of parents vs. 84.4% of child care workers appeared to be confident in sterility of PIF (P < 0.001). Statistically significant differences were also found between the mean scores of answers pertaining to washing hands before preparing PIF, sterilizing the equipment, using pre-boiled water cooled to no less than 70°C, immediate feeding, re-warming bottles after storage in refrigerator, throwing away reconstituted PIF after 24 hours, with the child care workers consistently being more likely to adhere to the practices suggested by WHO. In particular, a total of 64.1% of parents vs. 79.8% of child care workers agreed on the importance of dissolving PIF in water at no less than 70°C and, additionally, 86.1% of child care workers were likely to perceive risk of microbial growth in reconstituted PIF vs. 69.8% of parents (P < 0.001).

No significant differences were apparent between the mean scores of answers of the two groups to the questions pertaining to carefully cleaning bottles and nipples, refrigerating bottle feeds prepared in advance and dry and cool storing of PIF during its shelf-life. Of special interest, just 47.1% of parents and 50.7% of child care workers without statistically significant difference were likely to be aware of risk of burns to the infant's mouth associated to heating bottles in a microwave oven.

Additional analysis was conducted to identify differences in respondents' knowledge and self-reported practices on the basis of demographic characteristics of parental and child care worker respondents.

Answers to some questions by the parents' group were significantly impacted by age: in particular, younger parents were significantly more likely to be aware of presence of microorganisms in PIF (mean score 2.04 vs 2.21, P = 0.05), whereas the older ones were more likely to be aware of the importance of washing hands before handling feeding equipment (mean score 1.21 vs 1.31, P = 0.04), washing and rinsing thoroughly all feeding and preparation equipment (mean score 1.54 vs 1.88, P < 0.001), sterilizing the cleaned equipment (mean score 1.38 vs 1.56, P = 0.008) and immediately feeding the infant (mean score 1.73 vs 1.91, P = 0.02). When education level was compared, more persons with 8 years or less of education were found to be aware of the importance of dissolving PIF in water cooled to no less than 70°C (mean score 2.50 vs 2.05, P < 0.001) and never using a microwave oven to re-warm feeds (mean score 2.91 vs 2.49, P = 0.001), whereas more educated respondents were more likely to adhere to the WHO recommendation to quickly cool and refrigerate bottle feeds when prepared in advance (mean score 2.65 vs 2.98, P = 0.02) and store PIF in a dry and cool place until its use-by date (mean score 1.38 vs 1.63, P < 0.001).

The analysis revealed also a significant association between child care worker's age and the answers to some questions. The older respondents were significantly more likely to better know the importance of some hygienic practices such as washing and rinsing thoroughly all feeding and preparation equipment (mean score 1.16 vs 1.35, P = 0.02), sterilizing the cleaned equipment (mean score 1.27 vs 1.59, P = 0.003) and immediately feeding the infant (mean score 1.46 vs 1.78, P = 0.01). Older respondents were also more likely than the younger ones to know that PIF, once reconstituted, is a very good culture medium for many microrganisms (mean score 1.64 vs 2.04, P = 0.02).

Among the child care workers' group a significant association between answers and education level was evident for two issues: persons with 8 years or less of education were more likely to be aware that PIF is not a sterile product (mean score 1.78 vs 2.03, P = 0.03), whereas more educated persons were significantly more conscious of the need for cooling and refrigerating feeds when prepared in advance (mean score 2.40 vs 2.81, P = 0.04).

Education level and age were not significantly associated in either groups of respondents (P = 0.20 for parents and 0.26 for child care workers).

No significant differences existed between the mean scores of the answers among both parents and child care workers by number of children.

## Discussion

The data on parents and child care workers' knowledge about microrganisms in PIF suggest that both consumers and skilled food handlers are generally unaware that there is some risk associated with powdered formulas, because they are not sterile when purchased. Prevalences as high as 73.2% and 84.4% were detected among parents and child care workers, respectively, higher than those reported by other Authors [15,20]. This confirms the need for manufacturers to revise the instructions on labels and give correct advice to PIF handlers. Also, the awareness of paediatricians and other health professionals must be raised regarding potential risks of PIF in order to correctly inform parents and caregivers. Labiner-Wolfe *et al*. (2008) have previously stated that a proportion larger than 80% of mothers reported not having received instruction on formula preparation and storage from a doctor or other health professional.

Curiously, younger parents and child care workers with 8 years or less of education were less confident in sterility of PIF. Overall, studies on food safety are inconsistent with regard to the relationship between risk perception, age and socioeconomic status [21]. However, our results confirm some previous evidence that more educated consumers are less worried about food safety issues [22,23]. Moreover, though children are perceived to be more vulnerable to food-related risks, evidence that people with children are less confident about the safety of food is inconclusive [24].

Risk of foodborne illness in infants could be reduced when caregivers strictly adhere to hygienic practices, such as washing hands before preparing PIF, sterilizing the equipment, immediate feeding, re-warming bottles after storage in refrigerator and throwing away reconstituted PIF after 24 hours. Our investigation reveals that child care workers reported consistently safer self-reported practices than parents in this portion of the questionnaire. It seems likely that these differences could be at least partially explained by the regular food safety training given to all food handlers in Italy according to the Regulation (EC) No 852/2004. On the other hand, reconstitution and handling of the infant formula in a potentially contaminated domestic environment, poorly cleaning feeding bottles or poorly maintaining equipment at home by mothers of young infants may put them at risk of infection, particularly when they are aged less than five months, low birth weight or immunocompromised [17,25]. Moreover, both virtuous and hazardous behaviours relating to feeding bottles have been previously reported among parents [17]. This emphasizes that a large room for improvement is accessible to effective educational messages.

Safer options were generally chosen by both parents and child care workers when answering the questions about safe handling and cleaning procedures, but older survey respondents were more likely to report safer behaviours. Child care workers more frequently agreed with the recommendation to use water at no less than 70°C to dissolve PIF than did parents. Moreover, education level of parents significantly affected the answering pattern, with those with 8 years or less of education more frequently agreeing. This finding is consistent with those of other studies, in which lower education levels were reported to be associated with safer food handling behaviours [22,23]. On the other hand, it should be noted that this issue is to a some extent controversial. WHO and FDA recommend to use water close to boiling point, but other scientific organizations, such as the ESPGHAN Committee on Nutrition and the American Dietetic Association do not, because of possible adverse effects on physical stability and nutrient content of the formula [26]. Yet, most labels instruct to prepare the bottle feed with water at 40-45°C, whereas some others do not even mention the temperature, generically suggesting to use warm water.

Approximately 50% of both groups of respondents agreed with the recommendation to not use the microwave oven to warm reconstituted formula. Parents with 8 years or less of education had significantly better scores on this issue, further confirming previous findings suggesting an inverse relationship between education and the likelihood of adopting safer behaviours [22,23]. In any case, microwave heating appears to be a widely diffuse practice and minimization of risks of infant scalding by suggesting safe heating procedures could be a more realistic option [27].

Our study has some limits. Because of the non-probabilistic sampling, generalization of the results is limited. Selection criteria of attendance in municipal child care centres likely skewed our sample to a lower socioeconomic group. Self-administration of the questionnaire tended to select more educated parents. Ethnicity of respondents also was significantly slanted toward Italian persons. It is likely that these issues would deserve closer attention in the future studies on infant feeding in private and public settings.

## Conclusion

Infant formula needs to be handled in a way to reduce the chance of infection. Industry and regulators should play the primary critical role in reducing the risk of illness from consumption of PIF and ensuring that it is as safe as possible. Improvements should start with the manufacturing process. At present, it is unfeasible to produce sterile PIF; however, the risk of growth of hazardous organisms in prepared PIF can be minimized by adequately instructing caregivers of infants in both the institutional and home settings. Alternative sterile products must be considered for infants at higher susceptibility to infections. In this regard paediatricians and other health professionals should be made aware of the potential risks of PIF and provide scientifically sound information to child care givers. The public should be informed by targeted educational messages not only of the risks associated with PIF, but also of the guidelines for preparation, storage and handling. A better understanding of risk perceptions and behaviors could lead to more effective food safety education materials and messages.

## Competing interests

The authors declare that they have no competing interests.

## Authors' contributions

GC and CM had primary responsibility for study design, supervised the execution of the study, performed the final data analysis and wrote the manuscript. RMV and IG participated in the execution of the study, performed the preliminary data analysis and contributed to writing the manuscript. All the Authors read and approved the manuscript.

## Pre-publication history

The pre-publication history for this paper can be accessed here:

http://www.biomedcentral.com/1471-2431/9/75/prepub

## Supplementary Material

Additional file 1 **Table 2**. Respondents' attitudes toward PIF preparation, handling and administration according to the status of parent or child care worker. Click here for file
