# Supplementary material for: A survey on knowledge and self-reported formula handling practices of parents and child care workers in Palermo, Italy
Source: BMC Pediatr. 2009 Dec 10;9:75. doi: 10.1186/1471-2431-9-75 (PMC2796653; doi:10.1186/1471-2431-9-75)
Supplement: Additional file 1 — Table 2. Respondents' attitudes toward PIF preparation, handling and administration according to the status of parent or child care worker. [file 1471-2431-9-75-S1.DOC]

Table 2. Respondents’ attitudes toward PIF preparation, handling and administration according to the status of parent or child care worker.

|  | Strongly  agree | | Agree | | No opinion | | Disagree | | Strongly disagree | | Mean (SD) | | *P* |
| --- | --- | --- | --- | --- | --- | --- | --- | --- | --- | --- | --- | --- | --- |
| Statement | Parent  % | CCW* % | Parent % | CCW* % | Parent % | CCW*  % | Parent % | CCW*  % | Parent  % | CCW* % | Parent | CCW* |  |
| 1. PIF is sterile (does not contain microrganisms before opening) | 23.6 | 38.1 | 49.6 | 46.3 | 19.1 | 10.4 | 7.2 | 4.6 | 0.5 | 0.7 | 2.11 (0.86) | 1.83 (0.84) | < 0.001 |
| 2. It is important to thoroughly wash hands with soap and water before handling all feeding and preparation equipment | 76.7 | 79.1 | 21.2 | 19.3 | 1.7 | 1.2 | 0.2 | 0.1 | 0.2 | 0.1 | 1.26 (0.52) | 1.16 (0.40) | 0.02 |
| 3. It is important to wash all feeding and preparation equipment thoroughly in hot soapy water, by using a tooth brush to scrub the inside and outside of bottles and teats, and rinse thoroughly in safe water | 55.5 | 67.7 | 31.6 | 19.4 | 3.9 | 3.2 | 7.8 | 7.7 | 1.2 | 1.9 | 1.68 (0.95) | 1.57 (1.00) | NS† |
| 4. It is important to sterilize the cleaned equipment using a commercial sterilizer or a pan and boiling water | 61.7 | 74.4 | 32.9 | 21.7 | 3.4 | 3.5 | 1.9 | 0.3 | 0 | 0 | 1.45 (0.66) | 1.30 (0.55) | < 0.001 |
| 5. It is important to dissolve PIF by pouring the correct amount in previously boiled water cooled to no less than 70ºC | 24.0 | 41.7 | 40.1 | 38.1 | 15.6 | 7.9 | 18.1 | 10.6 | 2.3 | 1.7 | 1.21 (1.1) | 1.92 (1.03) | < 0.001 |
| 6. It is important, after checking the temperature, to feed infant immediately | 34.8 | 57.4 | 52.8 | 38.1 | 8.1 | 3.2 | 3.5 | 1.3 | 0.7 | 0 | 1.82 (0.78) | 1.48 (0.63) | < 0.001 |
| 7. It is important to throw away any feed that has not been consumed within two hours | 67.4 | 71.7 | 28.7 | 23.6 | 2.0 | 2.2 | 1.5 | 1.0 | 0.5 | 1.6 | 1.39 (0.65) | 1.37 (0.73) | NS† |
| 8. It is important that, if should need to prepare feeds in advance for use later, they should be cooled quickly and placed in the refrigerator | 22.4 | 34.9 | 29.5 | 24.8 | 9.8 | 7.8 | 26.0 | 20.2 | 12.3 | 12.4 | 2.76 (1.37) | 2.50 (1.45) | NS† |
| 9. It is important to re-warm feeding bottles, after removing them from the refrigerator, by placing them in a container of warm water | 17.7 | 35.6 | 46.5 | 32.4 | 11.2 | 7.2 | 17.9 | 17.0 | 6.7 | 7.8 | 2.49 (1.17) | 2.29 (1.32) | 0.001 |
| 10. Never using a microwave oven to re-warm feeds is important because they could scald the infant's mouth | 18.0 | 27.5 | 29.1 | 23.2 | 17.5 | 11.1 | 28.3 | 27.9 | 7.1 | 10.4 | 2.78 (1.28) | 2.70 (1.39) | NS† |
| 11. It is important to throw away any refrigerated feed that has not been used within 24 hours | 37.2 | 56.1 | 41.9 | 28.2 | 6.7 | 4.3 | 10.4 | 7.9 | 3.7 | 3.6 | 2.01 (1.09) | 1.75 (1.09) | 0.001 |
| 12. It is important to store PIF in a dry and cool place and consume it during its commercial shelf-life | 59.7 | 68.6 | 37.0 | 27.9 | 1.0 | 0.3 | 1.4 | 1.9 | 1.0 | 1.3 | 1.47 (0.69) | 1.39 (0.72) | NS† |
| 13. PIF, once reconstituted with water, is an excellent culture medium for many microrganisms | 27.5 | 51.0 | 42.3 | 35.1 | 22.5 | 9.1 | 7.3 | 4.9 | 0.5 | 0 | 2.11 (0.91) | 1.68 (0.83) | < 0.001 |

* CCW = child care worker

† NS = no statistically significant
